# Plasmodesmata Function and Callose Deposition in Plant Disease Defense

**DOI:** 10.3390/plants13162242

**Published:** 2024-08-13

**Authors:** Jingsheng Chen, Xiaofeng Xu, Wei Liu, Ziyang Feng, Quan Chen, You Zhou, Miao Sun, Liping Gan, Tiange Zhou, Yuanhu Xuan

**Affiliations:** 1 College of Biology and Food Engineering, Chongqing Three Gorges University, Chongqing 404100, China; jingshengchen@sanxiau.edu.cn (J.C.); verailgd@163.com (W.L.); ziyangfeng2024@163.com (Z.F.); chenquan0616@126.com (Q.C.); sunmiao4458@163.com (M.S.); ganmei790717@163.com (L.G.); 2 College of Plant Protection, Northeast Agricultural University, Harbin 150030, China; oxuxiaofeng@163.com; 3 College of Plant Protection, Shenyang Agricultural University, Shenyang 110866, China; 4 State Key Laboratory of Elemento-Organic Chemistry, Department of Chemical Biology, National Pesticide Engineering Research Center (Tianjin), Nankai University, Tianjin 300071, China; 9920230120@nankai.edu.cn

**Keywords:** callose, plasmodesmata, permeability, plant immunity

## Abstract

Callose, found in the cell walls of higher plants such as β-1,3-glucan with β-1,6 branches, is pivotal for both plant development and responses to biotic and abiotic stressors. Plasmodesmata (PD), membranous channels linking the cytoplasm, plasma membrane, and endoplasmic reticulum of adjacent cells, facilitate molecular transport, crucial for developmental and physiological processes. The regulation of both the structural and transport functions of PD is intricate. The accumulation of callose in the PD neck is particularly significant for the regulation of PD permeability. This callose deposition, occurring at a specific site of pathogenic incursion, decelerates the invasion and proliferation of pathogens by reducing the PD pore size. Scholarly investigations over the past two decades have illuminated pathogen-induced callose deposition and the ensuing PD regulation. This gradual understanding reveals the complex regulatory interactions governing defense-related callose accumulation and protein-mediated PD regulation, underscoring its role in plant defense. This review systematically outlines callose accumulation mechanisms and enzymatic regulation in plant defense and discusses PD’s varied participation against viral, fungal, and bacterial infestations. It scrutinizes callose-induced structural changes in PD, highlighting their implications for plant immunity. This review emphasizes dynamic callose calibration in PD constrictions and elucidates the implications and potential challenges of this intricate defense mechanism, integral to the plant’s immune system.

## 1. Introduction

Food production is an issue of great concern globally, and one key element influencing food security has been the frequency of plant diseases, which has prompted greater studies into plant disease prevention. Botanists have been studying disease resistance for nearly a century, and significant progress has been made in the field of plant immunity. For nearly two decades in particular, researchers have been integrating into and explaining the plant immune system. With advances in science and technology, a deeper and more thorough understanding of the plant immune system has been attained. Throughout ontogenesis, plants have evolved various mechanisms to resist pathogenic infections [1]. Central to this defense are the physical barriers encompassing the epidermis and cell walls, which effectively hinder pathogenic invasion. Subsequently, plants have evolved a dual-layer immune system characterized by mutual interactions, which is triggered by pathogen-associated molecular patterns (PAMPs), referred to as PAMP-triggered immunity (PTI) [2,3]. Typical immune responses to PTI include Ca^2+^ influx, ROS (reactive oxygen species) burst, protein kinase phosphorylation, MAPK (mitogen-activated protein kinase) pathway activation, and the generation of PR (pathogenesis-related protein) genes [4]. As a countermeasure to PTI, pathogens that successfully colonize the host release effector proteins to launch additional attacks on the plants, and effectors can obstruct the PTI. In response, plants have evolved a heightened defensive strategy called effector-triggered immunity (ETI) [1,5]. ETI is an accelerated and magnified PTI reaction that leads to disease resistance, usually producing a hypersensitive cell death reaction (HR) at the infected site [6]. However, as plant immune signaling pathways have been investigated more, it has become clear that PTI and ETI are not two immune pathways that operate separately, but rather that there are numerous interacting variables [7,8]. Notably, recent years have witnessed substantial research endeavors aimed at unraveling the intricacies of pathogenic infections and the underlying mechanisms of plant diseases [5].

**Figure 1:**
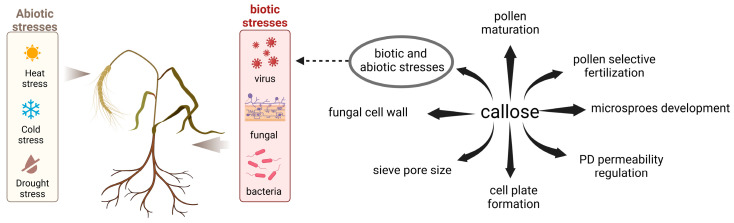
Significance of callose in the physiology of plants and fungi, including its role in pollen maturation, pollen-selective fertilization, gametophyte development, PD permeability regulation, cell plate formation, sieve pore size, and biotic and abiotic stresses process, and it is a component of fungal cell walls.

Callose, characterized as β-1,3-glucan, is synthesized during specific stages of plant growth and differentiation, exerting multifaceted functions across distinct plant sites (Figure 1). For example, callose synthesis and metabolism surrounding spores regulate the development of gametocytes [9,10]. Pollen maturation is aided by the transient buildup of callose in pollen tubes [10,11,12], with callose assuming an indispensable role in the selective fertilization of pollen, achieved through its deposition atop the pollen tube [13,14]. The accumulation of callose in the sieve tubes can alter the transport of substances in the tubes, affecting the growth and development of plants [15]. During cell division, callose is the predominant luminal component of the nascent cross-wall, transient deposition of callose occurs on the cell plate, and the primary cell wall or cell plate forms when callose builds up to a certain degree [9,16,17,18]. It aids in the targeted transport of chemical defenses, particularly at the plasmodesmata neck region, which exhibits resistance against pathogenic invasion and spread [19]. When plants are exposed to adverse environmental factors or pathogen invasion, the accumulation of callose is augmented as a defensive response to these unfavorable conditions (Figure 1). Beyond its pivotal role in plant cells, callose also plays a role in fungal cell walls. However, unlike in plants, callose is not typically a major component of fungal cell walls, which are primarily composed of chitin, β-glucans, and mannoproteins [20]. Earlier investigations have shown the involvement of callose synthase, β-1,3-glucanase, and PD-related callose binding proteins in regulating callose accumulation within plasmodesmata, thereby affecting their permeability [21]. Based on existing research, it is postulated that the quantity of callose deposited in the PD neck region exerts control over the pore dimensions, thereby regulating disease susceptibility.

In recent years, some authors have reviewed the regulation of plant immunity by callose and plasmodesmata. For instance, Wu et al. [21] and Amsbury et al. [22] examined the primary enzyme classes that modify callose levels and the impact of variations in callose deposition on PD pore size. Han et al. presented a complete explanation of the method by which callose-related proteins modify PD permeability by regulating callose accumulation, from the structure of PD formation to PD-related proteins [23]. The connection between callose and PD is now better understood, particularly how callose-related proteins modulate PD permeability by generating and degrading callose. Furthermore, the structure and morphology of PD are not static, and reviews of the structure and diverse morphologies of PD, as well as its significant role in plants, have been conducted [24,25,26]. Callose accumulation, a key reaction in plant immune response, is linked to plant disease resistance. Wang et al. described the plant immune signaling pathways involved in causing callose buildup and evaluated some evidence that callose modulates plant immunity [27]. Recently, German et al. studied the processes by which callose is regulated in plants, as well as the corresponding reactions of plants to PD and callose in plant-symbiotic relationships [28]. In this review, we summarize recent advances in understanding callose accumulation and PD structure in plant immune processes and highlight potential interconnections between these intricate mechanisms within plant immune processes, in addition to underscoring their significance.

## 2. Plant-Immunity-Related Callose Accumulation

### 2.1. Callose Accumulation as a Hallmark of Plant Immune Responses

Typically, conservative PAMPs can induce callose accumulation. Callose is deposited at the interface between the cell wall and plasmalemma, thereby altering the state of the cell wall. Upon pathogenic infiltration, the modified cell wall not only serves as a physical barrier against pathogens but also hinders the dissemination of toxins produced by pathogens.

Dating back to the 20th century, researchers discovered that the fungus *Colletotrichum lindemuthianum*, along with the bacterial pathogens *Pseudomonas syringae* pv. and *Xanthomonas campestris* pv., have the capacity to modify the structure of plant cell walls, thereby fostering the formation of papillae structures [29,30]. Immunogold labeling has revealed a direct correlation between callose, callose synthase, and papillae produced as part of the plant’s immune response. Consequently, callose and callose synthase emerge as integral constituents of the papillae structure [31]. This marks the initial highlight of the role of callose in the plant immune response. Nonetheless, subsequent findings challenged this notion, as papilla formation remained unaffected in callose-deficient and formation-impaired *pmr4* mutants, signifying the presence of components within the papilla structure beyond callose [32]. In modern methodologies, callose can be effectively visualized through aniline blue staining, generating a distinct blue hue that is observable under a microscope. This staining technique is the prevailing approach for callose visualization. Importantly, it not only facilitates the observation and localization of callose distribution but also allows for quantification through software tools such as Photoshop (version 23.0.2) or ImageJ (version 1.5.3). This integrated approach provides a convenient avenue for investigating the intricate dynamics underlying callose biosynthesis and regulation [33].

Evidence underscores a direct nexus between Flg22-induced immune response and callose accrual in Arabidopsis, notably demonstrated through augmented callose buildup in Arabidopsis leaves treated with Flg22 [34]. Processes such as oxidative burst, callose deposition, and the accumulation of pathogenesis-related proteins (PR) are shared facets within defense-related reactions. The accumulation of callose is influenced by environmental variables and is linked to other immune pathways, akin to other immune responses. The extent of callose accumulation manifests as modulation upon the pathogen-triggered induction of systemic acquired resistance (SAR) in plants. Arabidopsis possessing SAR exhibits a heightened callose deposition [35]. Salicylic acid (SA) can initiate SAR in plants and is emerging as a pivotal signaling molecule in plant immunity. The accumulation of callose in SA-treated plants exhibited a notable increase compared with pre-treatment levels [35]. This observation strongly implies the ability of SA to stimulate callose production. The role of SA as a signal in the plant immune response is widely acknowledged and intertwined with the pivotal activity of callose accumulation in PTI. Notably, SA, acting as the foremost signal for plant defense, affects the regulatory mechanisms governing callose synthase. This phenomenon is exemplified by the post-treatment response in Arabidopsis, where the external application of SA triggers the activation of multiple callose synthases, resulting in the coincidental augmentation of callose accumulation [36]. Conversely, mutants deficient in SA biosynthesis display diminished callose accumulation [37]. Furthermore, the abscisic acid (ABA) pathway contributes to callose buildup [38], with ABA-mediated callose deposition correlating with plant growth conditions [39]. Notably, apart from immune-related stress, callose accumulation also occurs in response to abiotic stress signals such as mechanical injury.

### 2.2. Enzymatic Regulation of Callose Deposition

**Figure 2:**
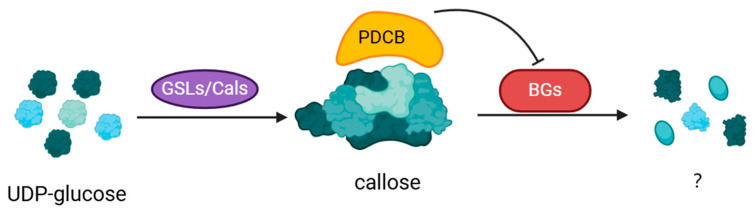
Enzymatic regulation of callose accumulation in plants. GSLs/CalS, enzymes presumed to facilitate the catalysis of 1,3-β glucan polymer synthesis from UDP glucose; BGs, responsible for the degradation of callose; PDCB, enhances the stability of callose through binding interactions.

Within the plant context, the intricate interplay between callose synthesis, degradation, and accumulation is precisely controlled by distinct enzymes. Key contributors include callose synthase (CalS), hydrolase (BG), and plasmodesmata callose binding protein (PDCB) (Figure 2). Their precise roles encompass neutralizing callose, thereby meticulously governing its accumulation patterns.

#### 2.2.1. Callose Synthase (CalS/GSL)

In the extensively studied model plant, *Arabidopsis thaliana*, a set of 12 GSL genes was identified, designated initially as GSL1–GSL12 [40]. An alternative annotation linked these genes to callose synthase genes, denoted as CalS1–CalS12 [41]. However, the serial numbering of these two gene identities did not align. The enzymatic function of callose synthase is attributed to the catalysis of the synthesis of a 1,3-β glucan polymer from UDP glucose, which serves as the pivotal driver of callose production. GSL genes bifurcate into two primary groups: (i) Contributors to fertility and cell division (GSL1, GSL2, GSL6, GSL8, and GSL10), where plausible redundancy is supported by experimental data. Notably, the callose synthases encoded by GSL1, GSL2, GSL6, GSL8, and GSL10 play essential roles in pollen development and are vital for pollen fertility and/or viability. (ii) Concomitantly, GSL5, GSL7, and GSL12 fulfill roles in augmenting structural cell wall integrity. The precise functions of the remaining four GSL genes remain elusive [42]. Mutant *cals5* underscores pollen sterility and impaired reproduction [9]. The CalS protein exhibits a multifaceted structure comprising the transmembrane, extracellular, and cytoplasmic domains. Notably, *Atgsl8* mutants display a distinct cytokinesis-deficient phenotype [18]. Earlier findings revealed that among Arabidopsis callose synthases, such as GSL5, GSL6, and GSL11, GSL5 deletion, in contrast to GSL6 and GSL11, obstructed callose synthesis. Remarkably, silencing GSL5/PMR4/CalS12 gene expression surprisingly resulted in augmented resistance to powdery mildew rather than susceptibility to the disease [43]. Notably, in *pmr4* mutants, the susceptible phenotype was restored by mutations in the SA pathway. Furthermore, callose synthesis and formation remained unaltered in the *pmr4* mutant under non-stressful biotic and abiotic conditions. This observation suggests that the influence of *pmr4* might be limited to the regulation of callose synthesis exclusively in plants facing adverse conditions. Notably, callose accumulation during normal plant growth and developmental processes remains unaffected by the pmr4 mutation [32]. These findings expand our understanding of the pivotal role of callose in plant biology. It is conceivable that most callose synthesis and degradation are regulated by either the plant immune pathway or one of the pathways associated with plant growth and development rather than by a simultaneous interplay between both pathways. Additionally, the overexpression of PMR4 significantly increased callose accumulation in Arabidopsis and barley, subsequently bolstering resistance to powdery mildew [44,45]. Unlike other GSLs, GSL7 is the only major gene responsible for callose synthesis within siliques, which is responsible for inflorescence development and silique transport in Arabidopsis, with a negligible influence on callose dynamics in other tissues and processes [46]. In addition, GSL12 and GSL8 are designated contributors to callose synthesis at the PD [47,48]. CalS1, which is found on the cell plate, is responsible for callose formation [17].

#### 2.2.2. Callose Hydrolase

Pioneering advancements in understanding callose hydrolase mechanisms and their interplay with disease resilience are exemplified by the case of GLU, which accentuates the susceptibility of tobacco to viral infestation [49]. Levy et al. identified β-1, 3-glucanase in the context of PD. This enzyme, encoded by the AtBG-papp gene, plays a role in callose degradation at PD sites, demonstrating co-localization with callose. Notably, T-DNA insertion mutants of AtBG-papp caused a reduction in PD permeability, effectively restricting the mobility of intercellular GFP [50]. β-1,3-glucanase belonging to the GH17 family in aspens operates under the mediation of gibberellin (GA) [51]. Moreover, the gene *GhGluc1*, encoding β-1,3-glucanase, was cloned and subsequently localized within cotton fiber. This gene was found to have a discernible association with both callose accumulation and degradation in the fibroblast structures. Intriguingly, the overexpression of *GhGluc1* resulted in an expansion of pore size in PD and concurrently led to a reduction in callose accumulation [51].

#### 2.2.3. Plasmodesmata Callose Binding Protein (PDCB)

In addition to callose synthase and callose hydrolase, a protein called plasmodesmata callose binding protein, encoded by AT5G61130, has been identified as a constituent localized within PD. This protein was fused to YFP and subsequently expressed in tobacco. Its subcellular localization was observed using fluorescence microscopy, which revealed a punctate distribution within the cell wall. This localization closely coincided with the presence of aniline blue-stained callose. Notably, the distinct fluorescent puncta retained this pattern even after plasmalemma separation, indicating the association of PDCB with the cell wall. Remarkably, immunogold labeling results also demonstrated the definitive localization of PDCB in PD. Through gel-blocking assays, the X8 structural domain of PDCB was found to exhibit a specific binding affinity for callose. In addition, green fluorescent protein (GFP) diffusion assays revealed that PDCB overexpression led to notable inhibition of GFP diffusion between cells. This finding strongly implies that callose deposition facilitated by PDCB modifies intercellular communication via PD, which is potentially attributed to the modulation of size exclusion limits (SELs) [52].

## 3. PD Is a Crucial Plant Component

PD constitutes channels formed by the plasma membrane crossing the plant cell wall, facilitating the interconnection between adjacent cells. This structure serves as a conduit for various plant symbioses [24]. As a pivotal communication conduit among plant cells, PD can regulate material transport and signaling pathways between plant cells. Furthermore, it possesses the capacity to modulate gene expression patterns and metabolic pathways within plants through the precise control of chemical compounds integral to signaling processes [53]. Meanwhile, PD also provides a pathway susceptible to infiltration by pathogens and viruses [54], thus intricately contributing to the plant defense architecture. PD exhibits dynamic responsiveness to numerous endogenous and exogenous factors, allowing for the continual adjustment of their permeability. Notably, callose deposition is a pivotal mechanism that crucially contributes to the regulation of PD permeability [26]. Empirical investigations conducted in Arabidopsis validated that PD can limit viral propagation within plants. This containment is achieved through the mitigation of permeability, facilitated by callose deposition at the neck of the PD [54].

### 3.1. Formation and Structure of PD

Primary PD is established as a consequence of endoplasmic reticulum connections, spanning microtubules within the cell plate during cytokinesis [55]. The intrinsic connection between callose and PD formation becomes evident as callose deposition occurs on the cell plate, concurrent with the inception of primary PD during cytokinesis. Subsequently, as cytokinesis is completed, callose degradation transpires within the cell wall, restricting its presence solely to the neck region of PD. This orchestration regulates the SEL of PD through variable levels of callose accumulation [26]. The evolutionary progression of PD involves the emergence of secondary PD from its primary counterparts on the cell plate. Various morphological classifications have been employed to systematically categorize the diverse morphologies and structural attributes exhibited by PDs. The simplest manifestation is an unbranched aperture that penetrates the cell wall. However, the spectrum of PD forms extends beyond this simplicity, encompassing diverse classifications such as the H type, Z type, and Y type, each delineated by distinct shapes. Furthermore, PDs exhibit a multitude of morphological adaptations, dictated by the specific growth and developmental requirements of the plant [16]. PD, particularly that between companion cells (CCs) and sieve elements (SEs), exhibits discernible molecular attributes. Notably, in PDs extending from SE to CC, a remarkable phenomenon transpires: the emergence of several finer branches from a primary, larger-pore PD. This architectural configuration resulted in a larger SEL on the SE side and a correspondingly smaller SEL on the CC side. This specific arrangement is one of the distinctive structural hallmarks characterizing PD situated between the SE and CC pairs [56]. Furthermore, the abundance of PD is subject to fluctuations in different phases of plant growth and development. For instance, PD undergoes reduction and ultimately disappears as a consequence of cellular differentiation during the maturation of guard and germ cells [57].

### 3.2. Callose Changes Permeability of PD

Functioning as conduits for a diverse array of macroscopic and microscopic molecules, including sugars, ions, amino acids, transcription factors, RNA, proteins, and various RNA-protein complexes, PD also regulates the transport and signal communication of intercellular substances [58]. A notable feature of PD is its capacity to facilitate the passage of non-cell-autonomous proteins (NCAPs), as corroborated by evidence reported in [59]. Crucially, PD is a dynamic entity adept at modulating its properties in accordance with the evolving requirements of the plant. Its capacity to transport substances exhibits variability and is intricately linked to the size of the SEL, a determinant of the efficacy of transport. Notably, SEL itself is a dynamic attribute subject to fluctuations [60]. The paramount determinant of SEL within PD is the accumulation of callose in the neck region, a process that is modulated by a range of proteins. As detailed in Table 1, callose synthesis is overseen by callose synthase, a phenomenon that contributes to the reduction in PD permeability. Conversely, the callose present in the neck of PD operates in an opposing manner: its degradation, catalyzed by callose hydrolase, facilitates the opening of PD channels [46]. Furthermore, PDCB plays a role in enhancing callose stability, subsequently leading to a reduction in the SEL of PD, despite its absence of direct involvement in callose production and breakdown [52]. In contrast to the first three, the PDLP class is primarily localized within the central region of PD rather than being restricted to the neck region. Studies conducted by [61,62] revealed that, upon the overexpression of PDLP5, Arabidopsis exhibited substantial growth abnormalities. In addition, PDLP demonstrated a nuanced ability to induce callose formation, leading to a reduction in the SEL of PD. Virus-encoded movement proteins (MPs) can modify the SEL in PD. Notably, investigations have demonstrated that PDMP can facilitate an enlargement in the PD pore size, thereby facilitating the passage of viral particles [63]. Plants employ a mechanism to detect external biotic or abiotic stresses, enabling the regulation of transmission efficiency through PD-linked cytoplasmic channels. Using fluorescent proteins or small molecules with inherent autofluorescence, researchers have conducted experimental measurements to quantify the diffusion rate of substances through the PD between cells. Carboxyfluorescein diacetate (CFDA) and GFP are the two most widely used markers for this purpose. CFDA, a small-molecule fluorescent dye, is capable of penetrating the cytoplasm and subsequently traversing the PD between cells, thus enabling the observation of its diffusion under a microscope. The innovative “drop-and-see” (DANS) test method, devised by Jung-Youn Lee’s team, serves to evaluate PD permeability. This approach simplifies the assessment of PD permeability by leveraging the properties of the CFDA [64,65].

### 3.3. PD’s Contribution to Plant Immunity

While PD serves as a conduit for plants to acquire nutrients and signaling molecules essential for growth and development, it can also be exploited by various organisms, including microbial pathogens, as a means to infiltrate and harm plants. The earliest instance of pathogenic incursion via PD was observed in the context of viral infections, and fungi have also been documented to exploit PD for dissemination. These insights indicate the intriguing possibility that pathogens might have evolved mechanisms to identify plant PD and infiltrate host cells, circumventing any deleterious impact on cell membranes and thereby evading the plant’s responsive mechanisms linked to stress-induced cellular demise. A recent study demonstrates that the Arabidopsis NOVEL CYS-RICH RECEPTOR KINASE (NCRK) is essential for the deposition of callose at PD in response to reactive oxygen species (ROS) stress. NCRK as a located PD protein regulates callose accumulation both under basal conditions and during ROS-induced stress. Furthermore, NCRK interacts with calmodulin-like protein 41 (CML41) and GSL4, highlighting its role as an upstream regulator in ROS-mediated PD closure mechanisms [71]. ROS serve as critical indicators in plant immune responses, underscoring the significant link between the callose-mediated regulation of PD permeability and plant immunity.

#### 3.3.1. PD’s Response to Viral Infestation

Although viruses possess considerably smaller genomes than other pathogens such as bacteria and fungi, their lack of a cellular framework renders them incapable of traversing the dimensions of the natural PD pore. In an ingenious adaptation, viruses have evolved a strategy involving the encoding of mobility proteins (MPs), enabling them to overcome this barrier and propagate seamlessly across cells via the PD. For instance, TMVMP has been documented to engage with ankyrin repeat-containing protein (ANK), thereby curbing callose accumulation within the PD neck region. This pivotal interaction amplifies the SEL of PD, augmenting the efficacy of viral mobility between cells. Similarly, CMVMP has been observed to break the microfilament structure of PD [70,72]. Recent investigations have revealed a complex network of interactions, including the engagement of PD-located protein (PDLP) with MPs, underscoring their pivotal role in mediating viral transit through PD [69]. The transfer of viral constituents between cells is further facilitated by intercellular filaments, a shrewd strategy employed by viruses to ensure their propagation. These filaments serve as conduits linking infected cells while strategically avoiding contact with the plasma membrane and circumventing disruption caused by the host’s secretory activities [73,74]. Notably, PDLP has been ascertained to function as an endogenous receptor protein, fostering the development of viral tubules [75]. Moreover, an intricate molecular interplay is observed, wherein TMVMP interacts with a protein involved in Ca^2+^ chelation. Although a direct demonstration of the significance of the interaction remains pending, its potential impact becomes evident through the substantial reduction in viral transmission achieved upon overexpression of the Ca^2+^-chelating protein [76]. This is because the Ca^2+^-chelating protein plays a critical role in maintaining the structural integrity of the plasmodesmata and regulating the flow of molecules between plant cells. Consequently, the stabilization of callose deposits at the PD neck, induced by reduced Ca^2+^ levels, may serve as a physical barrier, impeding the viral MPs’ ability to increase the SEL and facilitating the plant’s defense against viral movement.

#### 3.3.2. PD’s Reaction to Fungal Intrusion

In recent years, a compelling revelation has emerged concerning the rice blast fungus *Magnaporthe oryzae*, which uses PD as a conduit to propagate across cells during rice infestation. By employing live-cell imaging coupled with fluorescent probe confocal microscopy, researchers have illuminated the intricate process of fungal infiltration during the biotrophic phase. Remarkably, it was observed that upon traversing PD, the fungus’s invasive hyphae (IH) undergo a discernible reduction in size, closely conforming to the dimensions of the PD aperture [77]. Chitin is a polysaccharide widely present in the cell walls of fungi. When fungi infect plants, the plants can recognize chitin and activate their immune responses. Cheval et al. identified specific LysM receptor kinases, LYK4, LYK5, and LYM2, in plants that respond to chitin. Their research demonstrated that chitin signaling induces dynamic changes in these receptors’ localization and mobility within the plasmodesmal plasma membrane (PM). This response triggers the production of reactive oxygen species and callose, leading to PD closure, thus regulating callose accumulation and PD permeability [78]. Fungi conventionally deploy infection cushions or penetration pegs to penetrate the plant cell epidermis. This study indicates fungal sophistication in mounting attacks on host plants, as *M. oryzae* demonstrates a preference for propagation through PD, accompanied by adaptive size alterations, rather than opting for immediate extracellular invasion. This astute strategy deviates from traditional pathogenic incursions and underscores the ability of the fungus to exploit PD, despite its comparably restricted accessibility within the plant. Nonetheless, the precise mechanisms underlying the recognition and location of this minute entry point by the fungus remain unclear. Further exploration revealed the connectivity between IH and the location of effector proteins released by *M. oryzae* during rice cell infestation. Notably, the blast effector protein PWL2 was identified as capable of translocation to adjacent cells during infestation, established through the fusion of enhanced green fluorescent protein (EGFP) at its C-terminus. Although direct evidence of effector protein passage through PD is yet to be established, the intriguing association between PD and IH alludes to the potential of *M. oryzae* effectors to traverse PD [79]. Furthermore, it is widely recognized that fungal infections in plants result in the synthesis of toxins that disrupt typical cellular physiological processes. Regardless of their molecular size, these toxins easily diffuse between cells via PD transport, often more swiftly than the fungus itself. For example, typical cloud-like spots associated with rice sheath blight are often marked by discontinuous lesions. This intriguing phenomenon could be attributed to the propensity of much smaller toxins with lower molecular weights to relocate to new plant areas ahead of the mycelium, instigating fresh infections. Notably, the initial site of infestation retains the pathogen in the infected area due to a sequence of immunological responses such as cell necrosis [80].

#### 3.3.3. PD Response to Bacteria Infestation

In contrast to viruses and fungi, bacteria that infect plants predominantly operate within the extracellular matrix and do not require dissemination via intracellular routes. As a result, they circumvent the necessity of traversing PD and undergoing trans-cellular processes. However, PD indirectly plays a role in plant immune responses to bacterial infestations. Recent research has revealed the activity of bacterial effectors via PD and discovered that effector-induced plant immunological activity can activate the expression of PDLPs, which in turn inhibits PD permeability to limit the diffusion of effectors [81]. And in *Nicotiana benthamiana*, treatment with the 22-amino-acid peptide of bacterial flagellin (flg22) causes PD closure and inhibits the migration of a bacterial effector [82,83]. According to a recent study, Tee et al. discovered that flg22-like chitin acts as a key elicitor of immune response, and both of them can trigger PD closure via callose synthesis [83]. This process involves a convergence of signaling pathways at or upstream of callose synthesis, mediated by RESPIRATORY BURST OXIDASE HOMOLOGUE D (RBOHD). Furthermore, this study identifies PLASMODESMATA-LOCATED PROTEINS (PDLPs), particularly PDLP1 and PDLP5, and CALLOSE SYNTHASE 1 (CALS1) as central components in the response to microbial and salicylic acid (SA) stimuli. A crucial finding is the interaction between PDLP5 and NON-RACE-SPECIFIC DISEASE RESISTANCE/HIN1 HAIRPIN-INDUCED-LIKE protein 3 (NHL3), forming a complex that integrates multiple immune signals to activate CALS1 and induce PD closure [83]. On the other hand, bacteria employ effectors to infiltrate host cells, manipulating both callose levels and PD channels as part of their strategy to counteract the plant’s PTI response. This intricate interplay involves the regulation of callose and PD channels by bacterial effectors. Notably, the transit of these effectors between cells necessitates traversing the PD. The opening and closing of PD channels play a critical role in determining the severity of bacterial infestation within host organisms [30,81,84].

#### 3.3.4. Pathogen Manipulation of Callose and Plasmodesmata in Plant Defense Mechanisms

Plant pathogens interact with callose and plasmodesmata (PD) pathways, manipulating plant defense mechanisms to their advantage. *Pseudomonas syringae* induces callose deposition at infection sites, thereby reinforcing the cell wall to restrict pathogen spread [81]. Similarly, *Colletotrichum lindemuthianum* and *Erysiphe cichoracearum* trigger callose accumulation [84,85,86], enhancing structural barriers against invasion. The recognition of the flg22 peptide from *Xanthomonas campestris* by plants initiates callose synthesis [83], further bolstering the plant’s defensive barriers. Conversely, *Botrytis cinerea* secretes enzymes that degrade callose, weakening the cell wall and facilitating infection [87,88,89]. Pathogens such as *Phytophthora infestans* produce effectors that specifically target plasmodesmata, altering their function to promote pathogen dissemination [82,90]. *Hyaloperonospora arabidopsidis* employs effectors that modify PD permeability, disrupting intercellular communication and aiding infection [82]. *Magnaporthe oryzae* and *Cercospora nicotianae* modulate callose dynamics by inducing callose accumulation and inhibiting the induction of glucanases, respectively, playing crucial roles in the plant’s response to these pathogens [77,91]. The *Tobacco mosaic virus* (TMV) and *Cucumber mosaic virus* (CMV) utilizes its movement protein (MP) to target PD, enabling viral spread between plant cells, while *Rizoctonia solani* reduces PD permeability [69,70,72], benefiting the spread of pathogenic signals. *Ralstonia solanacearum* induces callose deposition and reduces PD permeability in plants, increasing PD permeability and facilitating its spread within plant tissues. These interactions underscore the diverse strategies employed by pathogens to manipulate callose and plasmodesmata, thereby modulating plant defense mechanisms. Understanding these interactions provides valuable insights into the molecular underpinnings of plant immunity and identifies potential targets for enhancing disease resistance in crops (Table 2).

## 4. Callose Controls PD Permeability for Plant Disease Resistance

**Figure 3:**
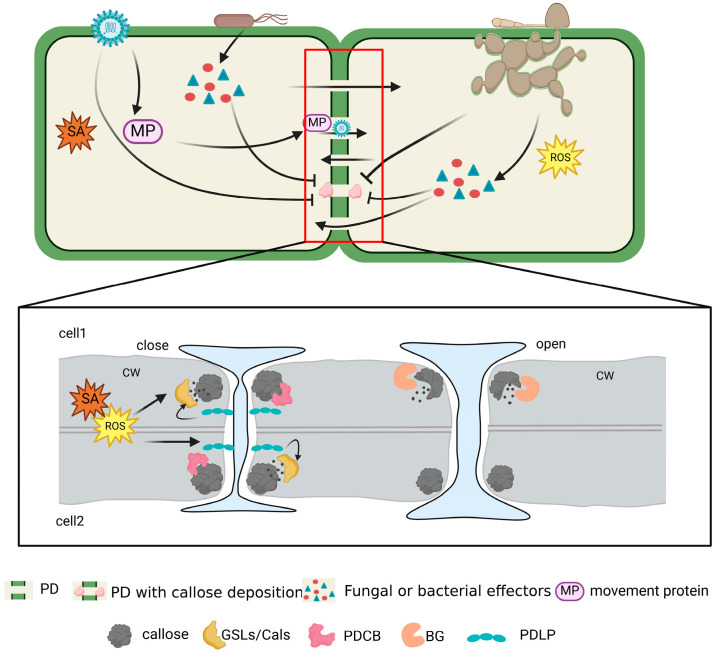
Dynamic responses of PD-related proteins to pathogenic intrusions: viral, fungal, and bacterial. MPs are known to facilitate viral particle transport via PD; when a PD presents a barrier to viral invasion in the host, the virus generates MPs specifically targeted at the PD, creating an aperture in the PD that allows the viral particle to pass through. Effectors secreted by bacteria and fungi during invasion of the host can travel through stomata to other parts of the host’s body. The distribution of effectors is strongly hindered when callose builds up in the PD neck region to decrease the PD pore size. Augmented callose deposition leads to PD constriction, thereby impeding the transit of bacteria, fungi, viruses, and their corresponding effectors.

The PD establishes a direct interconnection between adjacent plant cells, serving as a channel that bridges coplanar entities. Notably, callose plays a pivotal role in the nexus of PD, profoundly influencing its transit dynamics [92]. The intricate calibration of callose levels is controlled by an array of enzymes that indirectly regulate the transport capacity of PD to various chemical entities [20]. Figure 3 illustrates how viruses, fungi, and bacteria-infiltrating plants leverage PD to propagate their own structures and effectors. Concomitantly, when plants initiate a defensive response, they command the modulation of callose along with PD-related enzyme activities, resulting in the synthesis or degradation of callose contingent upon prevailing stress, thereby regulating PD permeability. The enzyme callose synthetase (GSL/CalS) plays a pivotal role in callose synthesis, an integral facet of a plant’s immune retort. Plant PTI triggers GSL/CalS expression. Furthermore, the signaling pathway initiated by the plant immune response concurrently stimulates callose synthesis through GSL/CalS. Additionally, the PDLP, a resident within PD, detects signals of pathogenic intrusion and consequently triggers GSL/CalS expression. In a concerted effort to hinder pathogenic infiltration and transmission, GSL/CalS promotes the accumulation of callose within the PD neck region, effectively constraining the PD aperture. However, this regulatory mechanism also engenders a trade-off, affecting the capacity of the plant to transport nutrients and relay signals. Consequently, when pathogens are sequestered within deceased cells or neutralized by host-released phytochemicals, the plant is compelled to restore the SEL of the PD channels. This restorative process necessitates the action of callose hydrolase, which dismantles the excess callose, thereby restoring the PD aperture to its original size. Notably, the callose-binding activity of PDCB slowed callose degradation and enhanced callose stability. However, a comprehensive understanding of their responsiveness to external biotic stressors remains a subject of ongoing investigation.

## 5. Conclusions

PD is a pivotal component of plant development, prompting numerous researchers to employ a multifaceted approach encompassing genetic, biochemical, and cell biology methodologies. This concerted effort has yielded the detection and study of several PD-related proteins and their roles over time. However, it is important to acknowledge that the intricacies in identifying PD constituents pose methodological challenges, constraining a comprehensive understanding of PD functionality. Emerging insights indicate a pivotal role of callose levels in the PD neck region, serving as a linchpin in the regulation of PD permeability and symptom pathways. Notably, an array of PD-related proteins, including GSLs, PDGBs, PDCBs, and PDLPs, among others, have been implicated in upholding callose homeostasis within the PD region. This review provides a thorough analysis of pertinent research reports and review articles. A brief description of callose homeostasis in plants and the mechanisms by which PD is regulated follow an introduction to the critical roles that callose and PD play in plants. Following that, the functions of callose and PD in plant immunity are examined, and lastly, the ways in which they control plant immunity are explained in regard to callose and PD’s interaction. We describe in detail how callose mediated alterations in PD pore size for several pathogens under biotic stressors.

**Figure 4:**
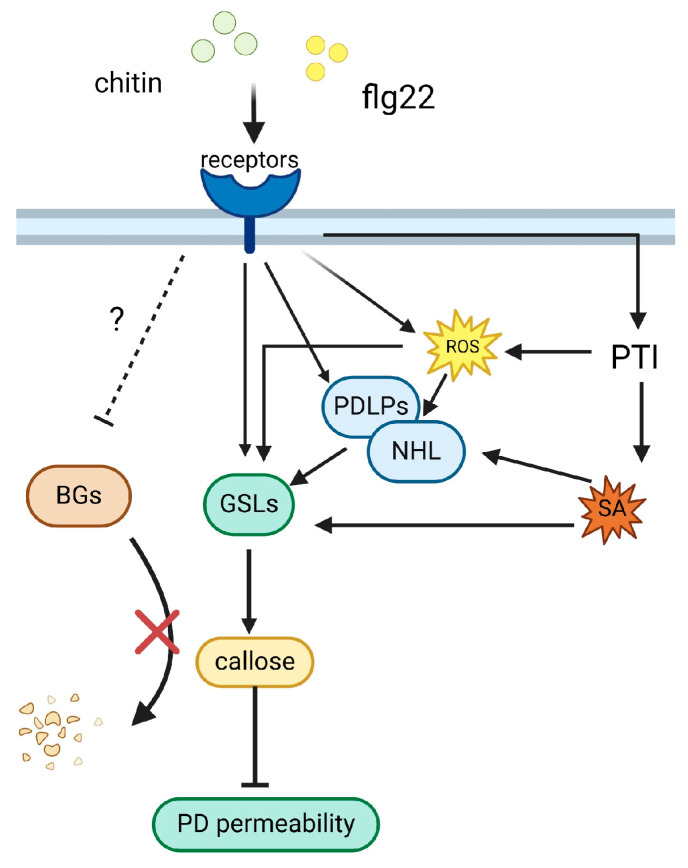
Signaling pathways regulating callose deposition and PD permeability upon fungal and bacterial infection in plants.

The intricate role of PD in plant disease defense is multifaceted and critical to understanding plant–pathogen interactions. PD as intercellular channels is pivotal in maintaining cellular communication and coordinating defense responses. During pathogen attack, the regulation of PD permeability becomes a key defensive mechanism. The dynamic deposition of callose, a β-1,3-glucan polymer, at PD is particularly noteworthy for its role in modulating these channels. Callose deposition is a rapid and reversible response to pathogen invasion, effectively isolating infected cells and preventing the spread of pathogens. This process is tightly regulated by a complex interplay of plant signaling molecules, including salicylic acid, jasmonic acid, and reactive oxygen species. These signals orchestrate the spatial and temporal deposition of callose, ensuring an efficient defense response while minimizing detrimental effects on cellular communication and overall plant physiology. The signaling pathways that trigger callose-mediated changes in PD permeability following fungal and bacterial infection in plants have been relatively well elucidated (Figure 4). The pathway indicates that upon activation by chitin and flg22, the receptors initiate a signaling cascade involving ROS, PTI, SA, and other signaling molecules. This leads to the activation of GSLs, resulting in the synthesis and accumulation of callose. Concurrently, PDLPs and NHL proteins modulate the permeability of PD, restricting pathogen movement and enhancing local immune responses. The dynamic regulation of callose synthesis and degradation, mediated by GSLs and BGs, respectively, is essential for the effective modulation of PD permeability. This balance ensures that while the plant enhance its defenses through callose deposition, it also maintains the necessary intercellular communication under non-stress conditions. Studies connected to callose and PD have become more lucid as research methods have improved. However, several questions remain unanswered, such as unraveling the mechanisms by which gene expression is regulated in response to particular developmental signals and environmental stressors. A common attribute of these PD-associated proteins is their transmembrane structural domains, which underscore their shared molecular characteristics. Subcellular localization analyses revealed typical PD-specific localization traits of these PD-associated proteins. Unraveling the mechanisms through which these proteins target PD has emerged as a critical endeavor in this context. The exact signaling cascades that trigger callose deposition at plasmodesmata, the potential cross-talk between different plant hormones, and the involvement of other cell wall components in this process require further investigation. Moreover, the balance between maintaining plasmodesmatal functionality for nutrient and signal exchange and sealing off these channels to block pathogen spread presents a critical area of study. Collectively, the spectrum of enzymes elucidated in this review intricately modulates the extent of callose deposition in PD. Consequently, the permeability of PD is controlled in response to diverse environmental conditions as well as biotic and abiotic stimuli. This dynamic control mechanism allows PD to adapt effectively to the surrounding environment.

## Figures and Tables

**Table 1:** Callose-related proteins and their corresponding functions.

Gene Name	Protein Function	Regulation of PD Permeability	References
GSLs/CalS	Callose synthesis	Reduce	[9,32,36,43,46,66]
BGs	Hydrolyze callose	Increase	[50,51,67,68]
PDCBs	Bind callose and enhance stability	Reduce	[52]
PDLPs	PD localization and regulate PD SEL	Reduce	[59,62,69]
PDMP	Enlarges plasmodesmata size	Increase	[63,69,70]

**Table 2:** Microbes and their interactions with callose and plasmodesmata in plant defense and immune responses.

Microbe (or Effector)	Interaction with Callose/Plasmodesmata	References
*Pseudomonas syringae*	Induces callose deposition	[81]
*Colletotrichum lindemuthianum*	Induces callose accumulation	[86]
*Xanthomonas campestris*	Flg22 triggers callose synthesis	[83]
*Botrytis cinerea*	Secretes enzymes that degrade callose	[87,88,89]
*Phytophthora infestans*	Effectors target plasmodesmata	[90]
*Erysiphe cichoracearum*	Induces callose accumulation	[84,85]
*Magnaporthe oryzae*	Induces callose accumulation	[77]
*Hyaloperonospora arabidopsidis*	Effectors alter PD permeability	[82]
*Cercospora nicotianae*	Blocks the induction of glucanases	[91]
*Tobacco mosaic virus* (TMV)	TMV movement protein (MP) targets PD	[69,72,73,92]
*Cucumber mosaic virus* (CMV)	CMV movement protein increases the PD size	[70,74]
*Ralstonia solanacearum*	Induces callose deposition and reduces PD permeability	[93,94]

## Data Availability

Data are contained within this article.

## Abbreviations

ABA	Abscisic acid
ANK	Ankyrin repeat-containing protein
BG	β-glucanase
CalS	Callose synthase
CCs	Companion cells
CFDA	Carboxyfluorescein diacetate
DANS	Drop-and-see
EGFP	Enhanced green fluorescent protein
ETI	Effector-triggered immunity
GA	Gibberellin
GFP	Green fluorescent protein
GSL	Glucan synthase-like
IH	Invasive hyphae
MPs	Movement proteins
NCAPs	Non-cell-autonomous proteins
PAMPs	Pathogen-associated molecular patterns
PD	Plasmodesmata
PDCB	Plasmodesmata callose-binding protein
PDGB	PD-associated b-1,3 glucanase
PDLP	PD-located protein
PR	Pathogenesis-related protein
PTI	PAMP-triggered immunity
ROS	Reactive oxygen species
SA	Salicylic acid
SAR	Systemic acquired resistance
SEs	Sieve elements
SELs	Size exclusion limits
